# Effects of Investment Experience on the Stock Investment Task: The Mediating Role of Risk Perception

**DOI:** 10.3390/bs13020115

**Published:** 2023-01-30

**Authors:** Zewei Li, Qi Wu, Pengfei Hong, Runzhi Tian

**Affiliations:** 1 Department of Psychology, School of Educational Science, Hunan Normal University, Changsha 410081, China; 2 Business Psychology, Business School, University of Leeds, Leeds LS2 9JT, UK; 3 Department of Management, School of Business, Hunan Normal University, Changsha 410081, China; 4 Cognition and Human Behavior Key Laboratory of Hunan Province, Hunan Normal University, Changsha 410081, China; 5 Finance Department, Business School, Durham University, Durham DH1 3LB, UK

**Keywords:** behavioral finance, irrational behavior, investment experience, investment behavior, initial risk perception

## Abstract

Due to the limitations of traditional financial analysis and the non-specificity of laboratory-based gambling tasks, it is difficult for researchers to isolate the independent contributions of risk perception and initial investment experience on novice investors’ behaviors. Thus, it is still necessary for researchers to describe the process by which investment experience affects the investment behavior of novice investors by employing the methods of psychological experiments that can control and eliminate these confounding variables in the laboratory. The current study created a stock investment task based on the balloon analogy risk task to simulate the stock market in the laboratory. Two hundred and twenty Chinese college students were recruited as participants. Chain intermediary model analysis showed that initial investment experience influences a novice investor’s behavior through risk perception. In addition, risk perception displayed the characteristics of continuity, in which the initial risk perception would affect later risk perception. These results indicate that investment experience does influence investment behavior. Different early investment experiences have correspondingly significant effects on the novice investors’ investment behavior through their risk perception. The results also suggest that novice investors can partly correct the effects of their initial investment experience through continuous feedback from the stock market.

## 1. Introduction

### 1.1. Two Contrasting Views on Whether Investment Behavior Is Influenced by Investment Experience

There are contrasting views on whether investors’ prior experience can significantly impact their subsequent investment behavior in the stock market [1,2]. This controversy is especially prominent in the investment behavior of novice investors, who have basic financial literacy but who are not good at using mathematical tools for stock analysis. Thus, they are more likely to take heuristic approaches [3]. Some researchers reported that they could not find a significant correlation between investment experience and investment behavior [4]. They proposed that novice investors are unable to learn from their investment experience since those investors make the mistake of “self-attribution bias” by attributing the causes of investment failure to chance and the causes of investment success to their own ability. This behavioral investment bias, called overconfidence, may make novice investors reluctant to update their investment strategies and prevent them from learning from their past investment experiences [5]. In addition, self-doubt, the opposite of overconfidence, has also been found to cause investors to doubt the validity of their investment experience. Researchers also proposed that it is exhausting for novice investors to make comprehensive and reasonable decisions in the stock market [6,7], which may lead the novice investor to follow other seemingly professional investors or make decisions in a random manner [8]. However, some studies also suggest that the investment experience of novice investors can significantly affect their investment behavior, making them more professional during the investment process [9]. They found that accumulating investment experience in the stock market can improve investors’ performance by surmounting the irrational biases in investment, such as overconfidence, the disposition effect, and the herd effect [10,11,12].

The reason why previous studies held different opinions on the relationship between investment experience and investment behavior is the complexity of the stock market. Since traditional financial methods are exclusively reliant on analyzing the historical trading data of the stock market (e.g., investors’ stock age and cumulative number of stocks traded) [13,14,15], it is hard for researchers to isolate the independent contributions of investment experience and control the irrelevant variables such as gender, risk tolerance, emotion, age, financial literacy, etc. [16,17,18,19,20]. Numerous confounding variables will covariate with the variables of interest, obscuring the interested effects [21,22,23]. Thus, it is still necessary for researchers to describe the process by which investment experience affects the investment behavior of novice investors by employing the methods of psychological experiments that can control and eliminate these confounding variables in the laboratory.

### 1.2. The Laboratory-Based Gambling Tasks: The Balloon Analogy Risk Task

To avoid the limitations of traditional financial analysis, in the current study, we modified the balloon analogy risk task (BART) to further examine people’s risk-taking behaviors in specific contexts. The classic BART paradigm is a computer-based task designed for measuring individual risk-taking tendencies [24]. In the BART, there is a vivid balloon on the computer screen, and the balloon becomes bigger after it is inflated by participants, with a monetary reward for each pump. Each balloon is set to explode at pseudo-random intervals, and every inflation will lead to an increase in the likelihood of the balloon exploding. Once the balloon explodes, all the accumulated money will be lost. If participants expect that the balloon will become bigger after the next inflation without exploding, they can choose to continue pressing the inflation button. On the contrary, if the participants expect that the balloon will explode after the next inflation, they can choose to end this round and enter the next round of balloon inflation. Risk-taking behavior in the BART is quantified by the average number of pumps delivered in the balloons that did not burst. In previous studies, researchers have employed the BART paradigm to explore the characteristics of participants’ risk-taking behavior. They study the effects of individuals’ age, reward style, substance use, and framing bias on their domain-general risk-taking tendencies [22,25,26,27,28]. In particular, the primacy effect was also found in the BART paradigm, where participants pumped the balloon (i.e., risk-taking behavior) far more boldly after a safe beginning than after experiencing incidental failure, since initial perceptions of high risk suppressed participants’ risk-taking behavior [29,30,31]. These general findings focused on risk-taking behavior in the general domain. It is interesting to evaluate these conclusions in a specific environment, such as the stock market.

The reason why researchers adore the BART paradigm is due to the fact that it has the following advantages: first, it creates a dynamic, iterative decision environment where the decision maker makes repeated decisions and receives relevant feedback (i.e., whether the balloon explodes or not) [32]; second, the risk and reward increase with each inflation, which is in line with the law of investment returns [32,33]; third, the researcher can adjust the likelihood of risk occurrence (i.e., the balloon explosion probability) to investigate how humans behave in a dynamic risk environment. For instance, researchers manipulated the initial risk perception of participants by modifying the balloon explosion time in the BART paradigm [32,34]; fourth, as a laboratory-based gambling task, it can control the confounding variables (e.g., information transmission efficiency, laws and regulations, and company annual report disclosure) to isolate the independent contribution of the interested variables in the laboratory. These advantages suggest that the BART paradigm has excellent performance in exploring the decision-making strategies of participants.

### 1.3. The Relationship among Initial Experience, Risk Perception and Risk-Taking Behavior

Although traditional financial analysis suggests the differential impact of investment experience on investment behavior, a link between investment experience and risk perception is thought to exist in the behavioral finance domain. Researchers in this domain emphasized the role of investors’ attitudes as a mediator in the relationship between financial knowledge (i.e., investment experience) and financial behavioral intentions (i.e., investment behavior) [35]. Governments and financial institutions pay more attention to the relationship among investors’ confidence, investment growth, and macro-economic growth. They believe that the risk perception of the public is as important as gold. As long as there is no massive panic among investors, stock market prices will stabilize and there will be no massive macroeconomic recession [36,37,38,39]. Psychological studies on general decision-making also help to bridge the gap from past experience to risk-taking behavior. Some studies argue that initial risk perception and later risk perception of participants play different roles in the risk-taking experiment. For example, people’s perceptions of changes in asset value are shaped by the initial price fluctuations of assets [32]. This means that initial experience may have an influence on risk-taking behavior by first shaping risk perception [29,30,31]. Furthermore, initial perceptions of high risk made participants less sanguine about expected return, and participants were more adventurous after a safe beginning than after experiencing incidental failure [29,30,31]. In addition, according to the theory of “need for cognitive closure” [40,41,42] and findings from Koscielniak et al. (2016) [29], the experience on the first three balloons in the BART had a lasting impact on the participant’s subsequent risk-taking behavior, which indicates the continuity of risk perception in the absence of accumulating sufficient experience. These studies in general decision-making, although not specific to the finance domain, still contribute to shedding light on how investment experience affects investment behavior, particularly highlighting the intermediary role that risk perception plays in the process.

## 2. The Current Study

### 2.1. Hypothesis Development

The research in behavioral finance indicates the influence of investment experience, especially the initial experience, on investment behavior. Furthermore, research in the field of general risk-taking behavior suggests the role of risk perception in forming risk-taking behavior [13,21,22,23]. In the present study, the following hypotheses were proposed: In the stock market, initial investment experience will significantly impact novice investors’ initial risk perception, which in turn affects their later risk perception and investment behavior. This implies that risk perception mediates the relationship between investment experience and the investment behavior of novice investors in the stock market.

To avoid the limitations of traditional financial analysis and the non-specificity of laboratory-based gambling tasks, we created a new stock investment task (SIT) to simulate the stock market in the laboratory (by modifying the BART) to reveal the relationship among investors’ investment experience, risk perception and investment behavior [24,32]. The current study set up three groups with different financial risk exposures to manipulate the differential investment experience. Two stock risk probability assessments were used to generate data on initial risk perception and later risk perception. To describe the concrete path of how investment experience influences investment behavior, a chain intermediary analysis was established.

### 2.2. Simulating the Stock Market Using SIT Paradigm Based on the BART Paradigm

Here we compare the main features of the BART paradigm and the stock market, and argue that the stochastic structure under the BART paradigm is similar to the stock market in terms of exit mechanisms, gain mechanisms, and learning mechanisms. We also propose that the BART paradigm can be modified to be a valid tool for simulating the stock market in the laboratory.

**Exit mechanism.** The exit mechanism refers to the way in which stock investors terminate their current investment behavior and reap the previously accumulated returns. In the stock market, stockholders are free to hold stocks or sell stocks (i.e., arbitrage) [43]. In the BART paradigm, participants are also free to choose when to end the task [32]. If participants expect that the balloon will become bigger after the next inflation without exploding, they can choose to continue pressing the inflation button, which is similar to the decision of individual investors in the stock market who expect the stock price to rise and therefore continue to hold the stock. On the contrary, if the participants expect that the balloon will explode after the next inflation, they can choose to end the current round and thus enter the next round of balloon inflation, which is very similar to situations in which individual investors expect the stock price to fall, so they decide to sell the stock at a high price in advance. In addition, a safety account and an investment account are set up in the BART paradigm. The money that participants decide to invest in each round goes into the investment account. The money in the investment account will be impacted by the loss occurring in the BART paradigm, while the money in the safety account will not be affected. When predicting an unacceptable risk, participants can give up on the continuation of inflation. Then they can collect all the token rewards accumulated from previous inflations in the investment account [32]. These settings in BART are also very similar to the situations in which investors have sold stocks at the right time in the stock market.

**Gain mechanism.** The gain mechanism is the way in which equity investors suffer losses and receive gains. In the stock market, the higher the risk taken by the investor, the higher the risk premium the investor receives [44]. Similarly, in the BART paradigm, when investors successfully inflate a balloon, they will receive gains, and every inflation will lead to an increase in the likelihood of the balloon exploding. Once the balloon explodes, the investment account will lose all the returns, thus increasing the likelihood of a risk occurrence [32]. Therefore, the gain mechanism of BART is also very similar to the gain mechanism of the stock market.

**Learning mechanism.** The learning mechanism refers to the process by which investors change their investment behavior through feedback on their investment decisions. In the stock market, investors have to decide when to sell or continue to hold stocks. These investment behaviors will bring returns and risks, and each stock market opening provides feedback on investors’ investment decisions [45,46,47]. Investors have to predict the future trend of stocks according to changes in stock prices, and they also have to diagnose the irrational factors appearing in their investment process by self-reflection and summarizing their experience [48,49], which indicates that stock market investors have a learning mechanism. Similarly, in the BART paradigm, participants also need to assess whether there is a risk of balloon explosion in the next inflation and make a judgment on the trend of this risk [32]. The result of loss or benefit from each inflation brings investors successive feedback and stimulation, and the tension and excitement brought by this feedback is also similar to the feeling brought by the stock market to the stockholders [23].

It is worth noting that the BART paradigm, as a general approach for the study of risk-taking behavior, cannot be directly applied to investigate specific conditions of investment in the stock market [21,24]. However, it is a common understanding that risk-taking behaviors have the characteristics of being both domain-general and domain-specific [50,51]. By investigating risk-taking behavior in the general domain alone, it is hard to know whether novice investors possess similar behavioral patterns in specific domains (especially when we are trying to invest in some stocks) [50,51]. Thus, while the BART paradigm is transferable to stocks, it is still inadequate for us to use it to simulate the stock market, especially in simulating the gain mechanism of the stock market. However, it is also inappropriate for researchers to solely use financial methods to describe the risk-taking behavior of novice investors based on stock market transaction data alone. The current study still needs to illustrate the relationship among investment experience, risk perception, and investment behavior of novice investors through modified laboratory-based gambling tasks dedicated to the stock market [52]. To better understand the psychology of stock investors based on the classic BART paradigm, the current study created the SIT, which is specifically designed to simulate the characteristics of the stock market (see Section 3.1 for details).

## 3. Method and Materials

### 3.1. Participants

The required sample size was estimated using a power analysis based on the effect size of the correlation between risk perception and risk-taking behavior (analysis: multiple linear regression with four predictors) and a desired power of 0.99 (mean *r* = 0.33, α = 0.05) [50]. These parameters resulted in a goal of 216 participants. We recruited 220 participants *(M*_age_ = 21 years, *SD*_age_ = 1.2, 83 females and 137 males) from Hunan Province, China. The current study is intentionally oversampled to avoid the possibility of failing to meet the desired power due to invalid data. All participants were in full-time education at the business school and had primary financial knowledge but no practical investment experience. We recruited from the participant pool of Hunan Normal University by means of recruitment advertising. This participant pool can provide participants with homogenous socioeconomic status [53]. Participants were randomly assigned to one of the three different experimental conditions. Upon arrival at the laboratory, the participants were required to provide written informed consent and fill in their demographic information. Participants were required to complete all tasks in the laboratory and earned monetary rewards as incentives based on tokens for their performance in the experiment. This study was carried out in accordance with the recommendations of the Research Ethics Committee of Hunan Normal University, with written informed consent from all participants. All participants gave written informed consent in accordance with the Declaration of Helsinki. The protocol was approved by the Research Ethics Committee of Hunan Normal University.

### 3.2. Stock Investment Task

SIT is a modified BART paradigm for quantifying the risk-taking behavior of investors in the stock market. With the SIT, researchers can manipulate the stock market situation (e.g., a high-risk stock market or a low-risk stock market) and describe the level of investment of the participants. The differences between the SIT and BART paradigms will be presented in the following four aspects: bubble, risk type, principal, and stock risk probability.

**Bubble.** To enable participants to better understand the SIT as a simulation of the stock market, the balloon in the BART paradigm is presented as a bubble in the SIT, which is employed to indicate the stock price. The investment behavior of pressing the button of inflation is presented as continuing to hold the stock and entering the next trading day. The bubble becomes bigger after it is inflated, which means the stock price rises [24]. The bubble leakage represents a partial drop in the stock price, while the bubble explosion represents the situation in which the stock price deviates greatly from its real price and investors lose all of their invested assets. In the last case, the balloon may not change even after the participants press the bottom. The value beneath the bubble represents current stock prices, indicating the number of times the bubble has been effectively inflated (i.e., the number of keystrokes of the participants minus the number of bubble leakage; the experimental procedure scripts for stock investment task as well as the scripts for the statistical analysis can be downloaded from https://osf.io/wy847/?view_only=3a6d95264f68487f949bef98abd7f5de). In addition, instead of employing the adjusted BART scores as in the BART paradigm [29], the adjusted SIT score is employed to describe investment behavior. This score is calculated as the average number of days to hold the stock without a loss occurring.

**Risk Type.** The fluctuations in the stock market rarely make individual investors lose all their money, but the core feature of the return mechanism of the BART paradigm is “all-lose or all-win”: once the balloon explodes, the participants’ previous inflated returns will be emptied [32], which is not in line with the stock market and will easily make the participants risk averse and affect the accuracy of the experiment [54]. In accordance with the gain mechanism of the stock market, the consequences of each inflation in the BART are modified into four scenarios in the SIT: “gain”, “no gain and loss”, “partial loss”, and “loss of all gain”, “Partial loss” is set to three types, namely 25%, 50%, 75% loss, and “loss of all gain,” which is set to a loss of 100%. “No gain and loss” represents invalid inflation, simulating the situation in which the stock price remains the same as the last trading day [55]. Instead of “all-lose or all-win,” only one of the five outcomes will be presented after each inflation, thus reducing the interference of risk aversion and increasing the credibility of the task.

**Principal.** Participants are free to choose how much capital to invest, and the more capital they invest, the greater the returns and risks will be. Participants are given tokens as principal in each round and are asked to decide how much to invest in the stock. In order to ensure the successful implementation of the game, the current study set a minimum investment to force each participant to join in at least one round of the BART task. As Equations (1) and (2) show, participants invest a minimum of 1 unit of tokens but no more than 100 units of tokens, and the final return is reduced by the principal invested. High principal investments can partly reflect the high risk-taking tendency of investors. Therefore, the invested principal is also employed to measure the investment behavior of individual investors in the SIT. Participants were given 100 tokens as principal in each round and were asked to decide how much to invest in the stock. More principal investment would result in more gains when the stocks rise and more losses when the stocks fall. The formulas for calculating the return were as follows:

(1) $$i>0,\;R_{t}=\left(X+i-m\right)\left(1-P_{t}\right)-X$$

(2) i>0, Rt=(X+i−m)(1+0.002X)−X ; i=0, Rt=0

(3) $$TR=\sum_{t=1}^{30}R_{t}$$

where *R_t_* is the return of round *t*, *P_t_* (*P* = 0.25, 0.5, 0.75, 1) is the level of loss, *X* (0 < *X* <= 100) is the number of tokens chosen by the participants to invest at the beginning of each round; *i* denotes the number of trading days the participants hold the stock; *m* represents the total number of trading days in which the stock price does not change; and *TR* is the total return of the participants in 30 rounds of stock investment tasks. Equation (1) shows the gain calculation method when loss occurs. Equation (2) represents the gain calculation method when participants sell stock before the stock market bubble bursts. Equation (3) shows the calculation of the total return.

**Stock risk probability.** Similar to the BART, the probability of loss in each trial of the SIT is expressed by the probability loss formula.

(4) $$p=\frac{1}{n-i+1}$$

where *p* represents the probability that the bubble will explode or leak (i.e., the probability of loss), *n* denotes the maximum number of times a participant can inflate the bubble, and *i* represents the number of days the participants hold shares. Similar to the BART, the SIT will only instruct the participants that the bubble will be inflated after a certain time, and they can only invest according to their risk perception [30]. The same pseudo-random setting of pre-determined loss points and loss levels as in the previous study is adopted (see Supplementary Material) [50], and three random “unchanged” settings are added in each round of SIT. In the SIT, each bubble has a maximum capacity. Holding the stock beyond this capacity will result in an explosion of the bubble, and then it automatically moves to the next round of SIT. The risk of loss will be maximized beyond the maximum number of inflated bubbles, which means that a loss will definitely occur after the next inflation. Investors’ earnings will be deducted in part or in whole when a loss occurs. The manner of deduction is randomly set to four types, namely 25%, 50%, 75%, and 100%. Meanwhile, the current study also set the scenario of “no gain and loss”, which will randomly happen three times before the bubble explodes or leaks. But this scenario was not set in some trials where the explosion point was too far forward. The current study manipulated the loss points and loss levels for the first five bubbles by setting three experimental conditions in which different initial investment experiences were manipulated, as displayed in Table 1. Specifically, the loss points and the loss levels of the control-risk investment experience (CIE) condition were completely identical to the settings of the SIT in Table S1 of the Supplementary Material. The high-risk investment experience (HIE) condition employed earlier loss points and higher loss levels than the CIE condition, whereas the low-risk investment experience (LIE) condition had later loss points and lower loss levels than the CIE condition. The settings of the next 25 rounds of SIT in all experimental conditions were completely identical.

### 3.3. Stock Risk Probability Assessment Task

After completing the stock investment task, participants were required to assess the risk probability through a stock risk probability assessment task. In this task, participants would see a sequence of bubbles with 11 different sizes. For each bubble, its size was determined by the number of trading days it had been held, which was 0, 13, 26, 39, 52, 65, 78, 91, 104, 117, or 128 trading days, respectively. Then participants were asked to enter the probability from 0% to 100% to estimate the probability of loss occurrence (i.e., “partial loss” and “loss of all gain”) after continuing to hold stock at that specific bubble size. After completing the stock risk probability assessment, participants were debriefed and paid for their participation based on the tokens they had earned in the stock investment task [32].

### 3.4. Procedure

All tasks were performed using the PsychoPy 2020 software. In this study, participants were asked to complete two tasks: a stock investment task and two stock risk probability assessment tasks [32].

Participants were required to complete one SIT and two stock risk probability assessment tasks. Participants were asked to finish the SIT first. In the SIT, five items are presented on the screen: the bubble, the security account, the amount in the investment account, the current trading day, and the current stock price. In the SIT, there were 30 bubbles ready to be inflated. Therefore, there were 30 rounds in this task, and each round could only contain 128 trials at the most (i.e., the bubble would definitely explode after holding the stock for 128 trading days). To encourage the participants to hold the stocks for a longer time, the three events of “no gain and loss” were inserted before the loss point, which was randomly located, only when the loss point was set to be located after the 20th trial in a corresponding round of the SIT; otherwise, no events of “no gain and loss” were set. Participants could choose to sell the stock for a gain in each round of the task and move on to the accumulation of money, which would be stored safely in a secure account without being affected by stock market fluctuations. However, if the bubble leaked or exploded, with the computer playing the sound of an explosion or air leakage, the participant’s temporary account would suffer a pre-determined corresponding loss, and then the accumulated money in the temporary account would be stored in the secure account. Then the next round of the SIT was automatically initiated. Similar to the BART, the SIT only instructed the participants that the bubble would be inflated after a certain time and they could only invest according to their risk perception [30].

After finishing the fifth round of SIT (“R5 rating”), all participants were asked to pause the task and were then required to assess the risk probability through the stock risk probability assessment task to assess their initial risk perceptions. Then the SIT resumed. After completing the SIT (“R30 rating”), participants were asked to complete another stock risk probability assessment task again. Then participants were debriefed and paid for their participation based on the tokens they had earned in the SIT.

### 3.5. Data Analysis

All participants finished the tasks as requested, and no data were excluded from the analysis. The current study manipulated the participants’ investment environment through setting loss points and loss levels to generate three groups of participants who have different investment experiences as independent variables. Afterwards, we collected participants’ adjusted SIT scores and invested principal as dependent variables to represent investment behavior. Data from initial and later risk perceptions were created through twice-stock risk probability assessment tasks as the basis for chained mediating variables.

The present research adopted a sigmoid-shaped psychometric function to probe the perceived level of risk for each participant. This function is valid in a three-parameter item response model and is reinterpreted by giving the parameters new theoretical meaning in the domain of behavioral finance [32]. The results of the pilot study showed that the rating function provided a good fit to the subjective probability ratings (mean *R*^2^ = 0.98; see Supplementary Material). The function was given by

(5) $$p_{i\left(x\right)}=\gamma_{i}+\left(1-\gamma_{i}\right)\frac{1}{1+e^{-\frac{x-\mu_{i}}{\theta_{i}}}}$$

where *P_i(x)_* represents the subjective probability of a fall in stock prices on all trading days *x* for participant *i*. The *μ*, *θ* and *γ* are free parameters estimated from the data for each participant, describing different aspects of participants’ risk perception changes. The *μ* (the shift on the *x* axis) is the threshold parameter estimating the subjective probability of stock risk occurrence. The higher the value of *μ*, the lower the probability of risk estimation to hold the stock. The *θ* (the slope of the middle part of the curve) is the sensitive parameter; it controls the slope of the probability evaluation function, and describes how quickly the risk perception level switches from low to high. The smaller the value of *θ*, the faster the rise of the participant’s risk perception level. The *γ* (elevation of the curve) is the elevation parameter measuring the starting point (intercept) of the participant’s risk perception, and the larger the *γ* means the higher the participant’s initial risk perception. The separate curve for each participant was fitted by minimizing the squared deviations between the participant’s risk perception data and those predicted by the function. The present research fitted the data by using the optimize algorithm (i.e., the model function, optimize.curve fit) in Python and setting the ranges of the three free parameters as: *μ*: [−500, 500], *θ*: [−1, 100], and *γ*: [0, 1].

Although the correlation between risk perception and risk-taking behavior was identified in the general decision-making paradigm, it is not clear if it still exists in investment behavior. Therefore, a pilot study was conducted to investigate which of these three parameters of novice investors’ risk perception were related to their investment behavior. In the pilot study (power = 0.9, α = 0.05, 103 participants), participants were asked to complete two tasks: a stock investment task and a stock risk probability assessment task [32]. The correlation analysis showed that the threshold parameter μ had a moderately positive correlation with the adjusted SIT score (*r* = 0.48, *p* < 0.001). This result suggests that risk perception is negatively associated with investment behavior. Investors with higher levels of risk perception (a higher perceived likelihood of loss) may consider holding the stock for fewer days than those with more conservative estimations. The sensitivity parameter *θ* and the elevation parameter *γ* were not correlated with the adjusted SIT scores and invested principal, which means only the threshold parameter *μ* is able to denote risk perception and contribute to the change in investment behavior (see Supplementary Material Pilot study). Thus, the present research only used the threshold parameter *μ* to represent risk perception in this study.

## 4. Results

### 4.1. Psychometric Curve

**Figure 1:**
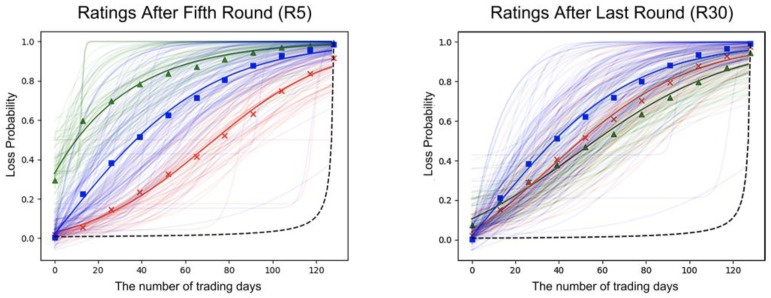
Fitted risk probability rating curves after the fifth round (R5) and fitted risk probability rating curves after the last round (R30). Note: The two panels showed the fitted risk probability rating functions for each participant’s probability ratings (light colored) based on the mean values for 11 estimates of the risk of continuing to hold stocks in the next trading day. R5 represents the fitted risk probability curves after participants completed the first five rounds of the SIT, and R30 after participants completed all rounds of the SIT. The three different colors of fitted risk probability rating functions in R5 and R30 represent three different initial investment experiences: The green curve (triangles) represents the HIE; the blue curve (square) represents the CIE; the red curve (exes) represents the LIE. The black dashed curve is the objective probability of the loss set by the experimenter based on Equation (1).

The fitted risk probability rating functions fitted the subjective probability ratings well (R5 CIE: mean R^2^ = 0.96, R5 HIE: mean R^2^ = 0.96, R5 LIE: mean R^2^ = 0.96; R30 CIE: mean R^2^ = 0.97, R30 HIE: mean R^2^ = 0.97, R30 LIE: mean R^2^ = 0.98). The fitted risk probability rating functions (R5) differed substantially depending on the experimental conditions. As shown in Figure 1, the results showed that the HIE group in R5 had higher estimates of the risk of holding the stocks than the CIE and LIE groups. These differences, however, were partly eliminated after completing the SIT (as shown in R30 of Figure 1), suggesting that participants with different initial investment experiences could converge their risk perceptions after going through the same stock market fluctuations. Meanwhile, Figure 1 also showed that the estimates of risk occurrence were higher than the objective probability in all experimental conditions.

### 4.2. Mediating Mechanisms

**Figure 2:**
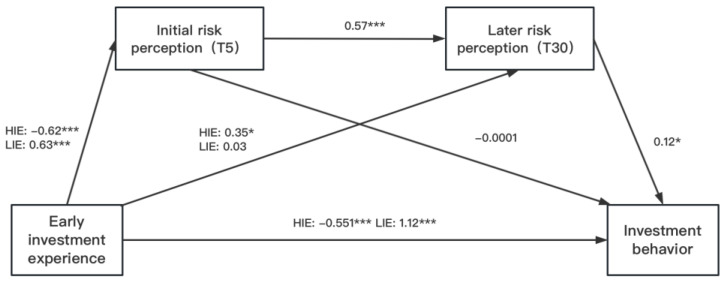
The chain-mediating role of initial risk perception and later risk perception between investment experience and investment decision and behavior. **Notes:** HIE, High-risk investment experience; LIE, Low-risk investment experience. * *p* < 0.1, *** *p* < 0.001. Coefficients in the Figure 2 are partially standardized because of the multicategory indicators of early investment experience.

The SPSS plugin Process 3.3 (Model 6) was used to perform the chain mediation model to evaluate the relationship between early investment experience (threshold parameter *μ*) and the adjusted SIT scores (only the data from the last 25 rounds of SIT were employed to calculate these two scores) [56]. The chain mediation model was constructed to investigate whether the differences in adjusted SIT scores between experimental conditions were caused by the differences in individual investors’ risk experiences and perceptions. Specifically, the current study examined whether the initial investment experience affects the adjusted SIT scores through the mediation of the initial and later risk perceptions (see Figure 2 for the conceptual model). The early investment experience was dummy coded. The CIE was set as the reference; therefore, HIE was coded as (1, 0) and LIE was coded as (0, 1). As shown in Figure 2 and Table 2, HIE negatively predicted (compared with CIE) initial risk perception (β = −0.62, 95% CI = [−109.51, −42.53]) and LIE positively predicted (compared with CIE) initial risk perception (β = 0.63, 95% CI = [41.06, 114.19]). Furthermore, no direct relationship was found between initial risk perception and adjusted SIT scores (β = −0.0001, 95% CI = [−0.02, 0.02]). The total effect of HIE and LIE on the adjusted SIT scores was significant (HIE: β = −0.551, 95% CI = [−11.41, −4.58]; LIE: β = 1.17, 95% CI = [13.30, 20.70]). The total effect of LIE on the later risk perception was significant (β = 0.39, 95% CI = [7.63, 90.22]). However, the total effect of HIE on the later risk perception was not noticeable (β = 0.003, 95% CI = [−37.75, 38.46]).

Further chain intermediary model analysis (see Figure 2 and Table 2 and Table 3) showed that initial risk perception and later risk perception could not independently mediate the relationship between initial investment experience and investment behavior unless they were considered as a whole. Specially, high risk experience negatively shaped initial risk perception, which in turn positively predicted later risk perception and adjusted SIT scores in the HIE group. On the contrary, low risk experience positively shaped initial risk perception, which in turn positively predicted later risk perception and adjusted SIT scores in the LIE group. The results suggested that because of the convergence of risk perceptions, the estimated error caused by different initial risk perceptions has been partially corrected. This supports the hypothesis that initial investment experience will indirectly influence investment behavior through investors’ initial and later risk perceptions.

## 5. Discussion

By employing the stock investment task (SIT) and stock risk probability assessment tasks in the present research, the current study investigated how stock market oscillation shapes novice investors’ risk perception, which in turn affects investment behavior. The results of the pilot study showed that the threshold parameter μ is a valid element that can be used to represent risk perception and predict investment behavior. A moderately positive correlation existed between risk perception and investment behavior, which was in line with the results of the general decision-making domain [32,57]. The results of the study showed the three groups with different risk experiences had different fitted risk probability rating functions. Finally, chain intermediary model analysis examined the validity of the overall pathway from investment experience to investment behavior and the co-mediating role of initial risk perception and later risk perception in this process. These results supported the hypotheses proposed in this paper. For novice investors, investment experience does influence investment behavior. Different early investment experiences have correspondingly significant effects on novice investors’ investment behavior through their perception of risk.

### 5.1. The Continuity of Risk Perception of Novice Investors

Expanding the work of Schürmann et al. (2019) [57], a chain intermediary model was employed in the present research to describe the individuals’ investment behaviors. The initial risk perception and later risk perception were entered as the chain mediators between initial investment experience and investment behavior. The results of this chain intermediary model showed that risk perception displayed the characteristics of continuity in which the initial risk perception would affect later risk perception [58]. This phenomenon was in line with the “need for cognitive closure” theory [40,41,42]. This theory suggests that, in the absence of sufficient data for inference, individuals who face ambiguity tend to hold a certain but inaccurate judgment for a long time to eliminate the stress from the ambiguous state. This indicates that novice investors may be reluctant to alter their judgment based on constantly updated information in the stock market [54,55,59,60]. The results in this paper suggest that the persistence of risk perception may be one of the manifestations of uncertainty and stress avoidance among novice investors.

### 5.2. Self-Corrected Risk Perception in Repeated and Dynamic Decisions of Novice Investors

The results also indicated that novice investors were unable to accurately perceive the probabilities of stock market risk and tended to overestimate the probability of loss. This may be due to the initial investment experience formed during the investment process interfering with the judgment of novice investors. Moreover, the risk perception was significantly affected by the initial investment experience in the early stage but not in the later stage. These results were consistent with previous findings of the irrational behavior caused by the “primary effect” (i.e., giving more weight to initial investment information) [61,62]. However, the findings also indicated that the investment decision bias caused by the differences in initial risk perception could be partly eliminated after completing the SIT, which suggests that the influence of the primary effect on investment behavior can be mitigated to a certain extent through cumulative feedback from the investment experience. These results corroborated the dynamic decision model [63,64], and the repetitive decision model [65] (These models suggest the important role of repeated and dynamic decisions in shaping risk perceptions and further suggest that the investment process can be decomposed into three stages: first, the formation stage, in which novice investors form risk perceptions based on their investment experience and investment environment [66]; secondly, the decision stage, in which investors make relevant investment decisions based on their perceived risk; and third, the feedback correction stage [67], in which investors obtain feedback from the results of their investment decisions [43,44] and re-adjust their investment behaviors by the accumulation of the investment experience [68,69]. The description of the investment process may help answer the question of whether novice investors can improve their investment performance through the continuous learning process [70]. This process also proposes that novice investors who have taken on risky fluctuations in the initial stage (e.g., bear or bull markets) use their strategies with caution (due to the continuity of risk perception). They need to remain sensitive to stock market feedback and be ready to adjust their investment strategies.

### 5.3. Limitations and Future Research

Although the present research offers certain theoretical contributions and practical implications, several limitations have been observed. First, although the risk stochastic structure of the stock investment task is comparable to that of the stock market, it still cannot cover all the characteristics of the stock market [15]. The impact of initial investment experience on investment behavior requires further evaluation by other researchers. For example, many people choose to trade stocks through brokers who get commissions from stock trading [71]. Previous studies found a reduction in risk aversion occurs when people are paid for making choices for others [72,73,74], suggesting that brokers may have a more complicated risk perception process than individual investors. Therefore, the stock investment task can be improved by adding a compensation system and integrating the self–others decision model to further explore the difference in risk perception between brokers and individual investors. On the other hand, since brokers, as investment veterans, have more sophisticated financial knowledge than novice investors, it would be beneficial to compare the investment process of an investment broker with that of a novice investor.

Second, to simulate the real risk environment of the stock market, the present research set up return formulas that reflect the positive correlation between risk and return. The investment behavior of novice investors manifests both their estimation of risk and return, while the novice investor’s risk perception only measures the likelihood of risk occurrence, which distinguishes risk and return as two variables. Previous studies have shown that the setting of rewards will have an impact on the participant’s risk propensity [26,75]. They showed that in the BART, participants who were paid for their performance displayed fewer risk-taking tendencies, while participants who were paid a flat-rate amount for just completing the experimental tasks tended to have more risk-taking behaviors because they had nothing to lose. The present study only used monetary rewards based on tokens for performance as incentives. Therefore, this setting may interfere with the formation of participants’ risk perception [76]. The present research still needs to take different compensation measures to verify the formation process of the novice investors’ risk perception in the future.

Third, researchers can also expand the graphology of simulated stock changes. This paper uses a bubble image as a risk cue, and others can use other graphs such as curves to simulate stocks, which reflects the flexibility of the SIT task. However, a wide variety of graphs have to characterize the core features of stock markets (e.g., return mechanisms, exit mechanisms, etc.).

Finally, numerous studies have discovered the important role of loss aversion in risk decision-making [77,78]. Therefore, risk aversion may also affect the formation of risk perception [79]. Other researchers can further discuss the role of loss aversion in the investment process from a behavioral finance perspective. Previous research has shown that different risk types will shape the corresponding risk perceptions [57,80,81]. Therefore, researchers can set different stock market curve types to simulate different stock market risks and observe changes in investors’ behaviors.

## 6. Conclusions

Investment experience does influence investment behavior. Different early investment experiences have correspondingly significant effects on novice investors’ investment behavior through their risk perception. Initial and later risk perception of novice investors mediate the relationship between investment experience and investment decision behavior. In addition, while novice investors tend to overestimate the probability of loss in the stock market, they are able to partly correct the irrational deviations caused by their initial risk perception experience through continuous feedback on their own investment decision results.

## Figures and Tables

**Table 1:** High risk and low risk loss points and levels.

Number	Loss Points	Levels of Loss	Flat 1	Flat 2	Flat 3
1	13/98	0.5/0.25	10	21	50
2	12/104	0.75/0.25	35	59	100
3	10/100	1/0.5	4	16	19
4	5/85	1/0.25	45	48	57
5	6/110	1/0.25	3	8	11

Note: The left side of “/” is the highest number of bubble inflation in the HIE condition, while the right side is the LIE condition. Flat 1, 2, 3 represent the three trading days in the LIE condition and CIE condition in which the stock price does not rise and fall.

**Table 2:** Partially Standardized Effect and 95% CIs for Direct and Indirect Effects.

Path	Effect	SE	95% CI
Total effect			
HIE→IB	−0.550 ***	1.73 ^a^	[−11.41, −4.58]
LIE→IB	1.17 ***	1.88 ^a^	[13.30, 20.70]
HIE→LRP	0.003	19.33	[−37.75, 38.46]
LIE→LRP	0.39 *	20.95 ^a^	[7.63, 90.22]
Specific indirect effect			
HIE→IRP→IB	0.0004	0.84	[−0.09, 0.14]
LIE→IPR→IB	−0.0004	0.80	[−0.11, 0.10]
HIE→LRP→IB	0.04	0.62	[−0.003, 0.16]
LIE→LPR→IB	0.004	0.17	[−0.01, 0.04]
HIE→IRP→LRP→IB	−0.04 ***	0.37 ^a^	[−0.10, −0.004]
LIE→IRP→LRP→IB	0.04 ***	0.38 ^a^	[0.004, 0.11]

**Note:** HIE, High-risk investment experience; LIE, Low-risk investment experience; IRP, initial risk perception; LRP, later risk perception; IB, investment behavior. * *p* < 0.1, *** *p* < 0.001. ^a^. Empirical 95% confidence interval does not include zero.

**Table 3:** Means and variances for each variable (*N* = 220).

Variable	M	SD
**CIE Condition**		
μ5	−9.42	114.61
μ30	−5.52	113.96
adj SIT Score	24.06	9.70
Principal	70.65	67.51
Profit	499.82	193.25
**HIE condition**		
μ5	−85.20	138.13
μ30	−5.16	170.66
adj SIT Score	16.07	9.81
Principal	56.87	23.36
Profit	390.74	137.78
**LIE condition**		
μ5	68.20	18.08
μ30	43.41	23.18
adj SIT Score	41.06	14.20
Principal	67.28	35.62
Profit	350.86	201.82

## Data Availability

The raw data supporting the conclusions of this article will be made available by the authors, without undue reservation.

## Supplementary Materials

The following supporting information can be downloaded at: https://www.mdpi.com/article/10.3390/bs13020115/s1, Table S1: List of loss points and levels, Figure S1: Fitted Risk Probability Rating Curves, Table S2: Means, variances and correlations for each variable in pilot study.
