# Supplementary material for: Effects of Investment Experience on the Stock Investment Task: The Mediating Role of Risk Perception
Source: Behav Sci (Basel). 2023 Jan 30;13(2):115. doi: 10.3390/bs13020115 (PMC9952433; doi:10.3390/bs13020115)
Supplement: Supplementary file 1 [file behavsci-13-00115-s001.zip › behavsci-2169405-supplementary.pdf]

1. List of loss points and levels

We adopted the same pseudo-random setting with pre-determined loss points and loss levels, and we added three random "unchanged" settings per stock investment task round. In some cases, the loss point was too low, thus we didn't set flat situation.

Table S1. List of loss points and levels.

Round	Loss point	Loss level	Flat 1	Flat 2	Flat 3
1	65	0.5	10	21	50
2	104	0.25	35	59	100
3	39	1	4	16	19
4	80	0.25	45	48	57
5	21	0.75	3	8	11
6	8	0.5	\	\	\
7	12	0.75	\	\	\
8	96	1	45	59	82
9	60	0.75	13	27	56
10	38	0.75	13	30	32
11	64	1	24	32	35
12	101	0.25	35	78	93
13	26	0.5	5	14	17
14	34	0.5	1	23	25
15	41	0.25	2	4	17
16	12	1	\	\	\
17	62	0.75	33	53	67
18	95	1	3	34	82
19	75	0.5	23	35	71
20	13	0.5	\	\	\
21	70	1	48	49	63
22	112	1	53	78	101
23	30	0.5	6	8	19
24	88	0.75	26	71	79
25	9	0.25	\	\	\
26	72	0.75	13	38	58
27	91	0.25	44	58	90
28	17	1	\	\	\
29	115	0.25	21	76	110
30	52	0.5	24	32	48

Table S1 showed the loss points (the number of possible pumps for each round), loss level, and flat situation (no gain and loss) of the control-risk investment experience (CIE) group. Loss points were taken from the original study by Lejuez et al (2003) [50]. As for the high-risk investment experience (HIE) group and the low-risk investment experience (LIE) group, we manipulated loss points and loss levels for the first five bubbles by setting two experimental conditions with different initial investment experiences (as shown in the formal study, Table 1).

Note: The "/" represents no flat in this round. Flat 1, 2, 3 represent the three trading days which the stock price does not rise and fall.

2. Pilot Study: Characteristics of novice investors' risk perceptions

Although the correlation between risk perception and risk-taking behavior was identified in the general decision-making paradigm, it is not clear if it still exists in investment behavior. According to the hypothesis, investment experience will affect investment behavior through risk perception, which requires a clear correlation between risk perception and investment behavior. Previous studies adopted a sigmoid-shaped psychometric function to probe the risk perception curve through a three-parameter item response model (i.e., threshold level, slope level, and elevation level) [32,57]. However, it is unknown whether this fitted function can be used in SIT. Thus, to demonstrate the correlation between risk perception and investment behavior, it is a primary necessity for us to ascertain the quantitative indicators that are appropriate for risk perception. The pilot study first tested whether this fitted function could be applicable to the SIT and investigated which of these three parameters of novice investors' risk perception were related to their investment behavior in the financial domain. Adjusted SIT scores and the invested principle were used to quantify investment behavior in SIT. The pilot study also explored whether individual investors can accurately perceive the occurrence of objective risk in the stock market. In the pilot study, data was generated by the stock investment task and the stock risk probability assessment task. The risk perceptions of novice investors were quantified through a psychometric function. Correlation analysis was used to reveal the potential relationships between risk perceptions and investment behavior.

2.1. Participants

The required sample size was estimated by using a power analysis based on the correlation between risk-taking behavior and risk perception (mean $r = 0.33$) and a desired power of 0.9 ($\alpha = 0.05$) [50]. These parameters resulted in a goal of 88 participants. The pilot study finally recruited 103 Chinese adults ($M_{age} = 20$ years, $SD_{age} = 0.6$, 48 females and 55 males) from Hunan Province, China. We intentionally over-sampled to avoid the possibility of failing to meet the desired power due to invalid data. All participants were in full-time education at the business school and had primary financial knowledge but no practical investment experience. In addition, the participants were recruited from the participant pool of Hunan Normal University by means of recruitment advertising. This participant pool can provide participants with homogenous socioeconomic status [53]. Upon arrival at the laboratory, the participants were required to provide written informed consent and fill in their demographic information. Participants were required to complete all tasks in the laboratory and earned monetary rewards as incentives based on tokens for their performance in the experiment. The pilot study was carried out in accordance with the recommendations of the Research Ethics Committee of Hunan Normal University, with written informed consent from all participants. All participants gave written informed consent in accordance with the Declaration of Helsinki. The protocol was approved by the Research Ethics Committee of Hunan Normal University. Particularly, it should be noted that all these participants in the pilot study did not participate in the formal study.

2.2. Procedure

Consistent with the formal study, all tasks were performed using the PsychoPy 2020 software on personal computers. Similar to the formal study, participants were required to complete one SIT and one stock risk probability assessment task. The pilot study used the same settings as in the CIE of the formal study (e.g., the return formula, loss types, exit mechanism, loss points, and loss levels).

2.3. Data analysis

All participants finished the tasks as requested, and no data were excluded from the analysis. The pilot study collected participants' adjusted SIT scores and invested principle as dependent variables to describe their investment behavior in certain investment environments, and employed the stock risk probability assessment task to generate the risk perception data. A correlation analysis was performed between the parameters of the risk probability rating function and the adjusted SIT scores to reveal the degree of association between risk perception and adjusted SIT scores.

3. Results and discussion

On average, participants had an adjusted SIT score of 21.75 (*SD* = 3.25). The results showed that the rating function (black curve) provided a good fit to the subjective probability ratings (mean $R^2 = 0.98$). They also showed that the estimates of stock risk increased with the number of days in which participants held stocks. As shown in Figure S1, the black curve was significantly higher than the black dashed curve, which indicates that the subjective probability of risk occurrence was higher than the objective probability of risk occurrence. Therefore, these results indicate that participants overestimated the probability of actual risk occurring in the stock investment task.

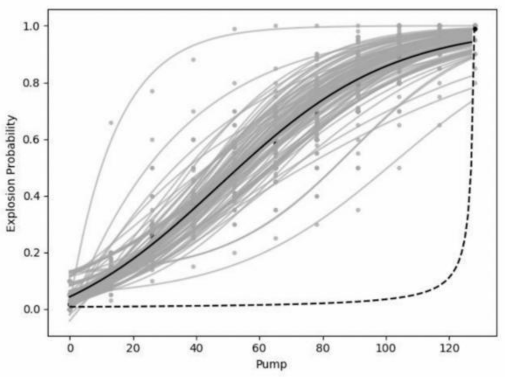

Figure S1. Fitted Risk Probability Rating Curves.

Note: the gray points indicate the data of 11 estimates of the risk of continuing to hold stocks in the next trading day for each participant; the gray curves indicate the fitted risk probability rating functions for each individual obtained by fitting the gray points; the black curve indicates the fitted function based on the mean ratings for each of the 11 rated bubble sizes; the black dashed curve is the objective probability of the loss set by the experimenter based on *Eq.*(4) in formal study.

The results of the correlation analysis are presented in Table S2. The threshold parameter μ had a moderately positive correlation with the adjusted SIT score ($r = 0.48$, $p < 0.001$). This result suggests that risk perception is negatively associated with investment behavior; investors with higher levels of risk perception (higher perceived likelihood of loss) may consider holding the stock for fewer days than those with more conservative estimations. The other two parameters, the sensitivity parameter θ and elevation parameter γ, were not correlated with the adjusted SIT score, which means only the threshold parameter μ is able to denote risk perception and contributes to the change in investment behavior.

Table S2. Means, variances and correlations for each variable in pilot study (*N*=103).

Variable	M	SD	1	2	3	4	5
1 μ	-15.66	138.50					
2 θ	24.21	7.87	0.03				
3 γ	-0.11	0.30	0.34*	0.004			
4 adj SIT Score	21.75	3.25	0.48***	-0.02	0.09		
5 Principal	85.13	18.01	-0.18	-0.09	0.001	-0.60***	
6 Profit	574.33	99.17	-0.02	-0.04	0.13	-0.28**	0.38***

Note: * represents the degree of significance. *$p < 0.1$, ** $p < 0.01$, ***$p < 0.001$.

The results (Table S2) also showed that the adjusted SIT score had a negative correlation with profit, and invested principal was positively correlated with profit, which indicates that a stock characterized by short-term trading was simulated by the stock investment task in the pilot study (i.e., the time interval between buying and selling was short). But the threshold parameter μ had no correlation with the invested principal. One possible explanation for this result is that in SIT, participants were encouraged to adopt short-term trading strategies, which might have changed the effect of the experimental return mechanism on the participants' risk perception and alleviated the participants' motivation to invest. The invested principle was not correlated with all components of risk perception, which is consistent with the work of Schürmann et al (2019) [57]. There are many different types of stocks in the stock market. Previous studies have shown that participants attempt to adopt targeted trading strategies for varieties of stocks to earn more profit. The pilot study described the trading strategy of novice investors in terms of trading days and invested principal. And the results showed that the SIT tasks employed in the pilot study research actually captured the characteristics of a stock market that encourages participants to adopt short-term trading. This may be caused by the beliefs held by investors that stock prices will not fall only in the short term [57]. Other researchers can further explore the impact of different types of stocks on individual investors' risk perceptions and trading strategies [41,42]. For example, researchers can use SIT to simulate a long-term stock characterized by long-term growth and encourage investors to adopt fundamental analysis and pay more attention to the future outlook of the stock.
